# Menopause and Crisis? Fake or Real: Comprehensive Search to The Depth of Crisis Experienced: A Mixed-method Study

**DOI:** 10.5539/gjhs.v6n2p246

**Published:** 2014-02-21

**Authors:** Nehle Parand avar, Leili Mosalanejad, Somaye Ramezanli, Fatemeh Ghavi

**Affiliations:** 1 Maternity Department, Research Center for Social Determinant of Health, Jahrom University of Medical Sciences, Jahrom, Iran; 2 Depertment of Mental Health, Research Center for Social Determinant of Health, Jahrom University of Medical Sciences, Jahrom, Iran; 3 Nursing Department, Research Center for Social Determinant of Health, Jahrom University of Medical Sciences, Jahrom, Iran

**Keywords:** menopause, hypochondriasis, psychosomatic disorder, crisis, qualitative research

## Abstract

**Introduction:**

Menopause is beyond the lack of menstruation and fertility decline in estrogen.

**Menopause is associated with at least three types of crisis:**

Biological, psychological and social. The aim of this study was to investigate psychiatric problems related to menopausal stress and experiences about psychological conditions related to menopause as a developmental crisis.

**Material and Methods:**

This mixed-method study (by triangulation approach) was done on 300 women in menopause age (44-54 years) by consensus sampling. Data gathering was from questionnaire conclude psychosomatic listed and hypochondria’s criterion that has been specified by DSMIV. The severity of the disorder was also collected by self-reported question. In the qualitative part, as a phenomenology study, data were gathered with Purposive sampling by a deep semi-structured interview. Data analysis was from content analysis).

**Results:**

Results showed that most of the disorders from psychosomatic listed experienced by women conclude: sexual problems 101(33.7%), hypertension 39(13%), and constipation 30(10%); 2.9% had experienced hypochondrias disorder. In the qualitative part, 5 themes were driven from the results of this study which described the structures of psychological experiences of the menopause as follows: change in emotion and mood, change in attitude, change in self-concept and change in interpersonal relationships.

**Conclusion:**

Menopause is a physiological process in women’s life, but due to many symptoms and complications, it requires culturally appropriate education, appropriate coping with problems and mental health promotion in this sexual crisis.

## 1. Introduction

Women comprise more than half of the population living on Earth. They are the most important basis of the family and the society; society’s health depends on supplying their health, cultural, and economic needs (Golchin, 2005). Women have a large number of duties whose accomplishment requires complete physical and mental health. Menopause is one of the most critical stages of the women’s life (Stwart & Baydo, 1993; Darren, 2004). Although women enter menopause with a lot of life experiences, they encounter various changes that they had not experienced before (Speroff, 2005). Menopause is defined as the permanent cessation of menstrual cycles for a year. Physiologically, menopause occurs due to the lack of mature follicles in the ovaries and subsequently reduction in estrogen secretion. The mean age of menopause is between 50 and 52 years (Speroff, 2005; Berek, 2007); however, it may vary from 44 to 54 years. Overall, by the improvement of life conditions and an increased life expectancy, more than one third of a woman’s life is spent in menopause (Scott, 1991). In general, menopause is accompanied by a large number of physiological and psychological changes. For instance, women may develop acute and chronic signs and symptoms resulting from lack of estrogen (Lichtman, 1991). These are called the climacteric symptoms and include hot flashes, atrophic changes of the genitourinary system, mental and physiological symptoms, cardiovascular diseases, osteoporosis, menstrual irregularities, mood changes (depression, anxiety, and irritability), changes in skin, memory loss, reduction of sexual desire, and sleeping disorders (Speroff, 2005; Berek, 2007; Scott, 1991; Ryan, 1997). Studies have also shown that lack of estrogen may increase the risk of depression (Blumel et al., 2000; Stirrat, 2000; Decherney, 2003; Shives, 2002; Birkhauser, 2002). For instance, Sagsozet et al. (2001) conducted a study using Beck’s inventory and showed that depression and anxiety had increased in the menopausal women. In addition, a study which was conducted on Iranian women using Beck and Kettle questionnaire revealed mild and severe depression in 32% and 5.3% of women, respectively. The study also showed a lower intensity of depression in married women and those who had some information about menopause (Rasoli et al., 2004). Moreover, Bungay et al. (1980) reported a higher percentage of depression signs in women referring to menopause clinics compared with the normal population. The study which was conducted on 2001 Australian women referring to health clinics showed that women with more hot flashes had higher physical and mental symptoms and a weaker health status.

Therefore, in order to increase conformity and understand its resultant changes, the knowledge of both women and their family members should be increased. Of course, it should be noted that menopause is not similar for all women but is affected by the individuals’ mental status, emotional health, and cultural and social background. Besides, Kaufert (1986) stated that women’s beliefs are effective in the formation of menopause experience. Since menopause (as a physiological crisis) is associated with stress, we expected that the disorder associated with stress as a psychosomatic disorder and hypochondriasis also can be seen more. Therefore, this study aimed to assess various psychological aspects of this crisis in menopause women as a mixed-method study (Olsen, 2004).

## 2. Materials and Methods

This study is mixed-method study by a triangulation approach. This method is used by qualitative researchers to check and establish validity in their studies by analyzing a research question from multiple perspectives. Triangulation Theory involves the use of multiple perspectives to interpret a single set of data. Unlike investigator triangulation, this method typically entails using professionals outside of a particular field of study (Olsen, 2004; Hsieh & Shannon, 2005; Streubert & Carpenter, 2007).

Due to the fact that psychological issues can be completely understood through qualitative studies, this study was also performed using a mixed-method study (Olsen, 2004) with a triangulation approach. Also, we used sequential triangulation in the study design. According to Morse (1991)” sequential triangulation is utilized when the results of one approach are necessary for planning the next method”.

In general, qualitative research provides a systematic approach for describing individuals’ experiences and in fact give meaning to these experiences (Polit & Beck, 2006).

The quantitative part of the present study is a cross- sectional study which was conducted on 300 menopausal women 44-54years during 1 month at February 2013 by consensus sampling. These women had referred to specialized clinics as patients’ companions and at least 6 months had passed from their last menstrual cycle. The reason for selecting this location was availability to a large number of samples simultaneously, so that it was possible to be included in the study. In this study, menopause was considered as the only stress experienced as a developmental crisis and individuals, who had experienced average and severe stress conditions based on Holmes and Rahe (1967) scale were excluded from the study. In order to investigate the effect of mental stressors on the people’s health in various age ranges, they developed a scale which predicts the risk of diseases considering the number and type of life changes during one year. In this scale, the intensity of life crises has been categorized as mild (150-199), average (200-299), and severe (above 300). According to this scale, a score between 11 and 100 was assigned to each crisis resulting from 41 life situations.

500 of 800 people were excluded from the sampling due to moderate to severe stressful experience, some physical and psychiatric disorders and 300 people who were stress from menopause condition only, or people who had experienced mild stress conditions during menopause were recruited. Each of the women within the sampling frame received fallowing self-report inventories (from a list of 13 condition symptoms and diseases) to indicate their severity of occurrence (high to a symptomatic) among those that they may have experiences during menopause period.

Data, obtained for investigating psychosomatic diseases, were analyzed using descriptive statistics (frequency and percentage). Also, in this sample, the rate of hypochondria disorders was evaluated. Based on DSM IV criteria “in spite of being physically healthy, these individuals assume themselves to be sick and complain about imaginary pains and duties. Although complementary medical examinations of these individuals do not show any problems, the hypochondriacs believe in a serious fatal disease. Hypochondria are accepted as a disease if 6 months have been passed from its onset (Sadock, 2007).

Considering the importance of deeply investigating individuals’ experiences, the subjects were selected through purposive sampling in the current qualitative study (phenomenology). The research community included all menopausal women who had referred to specialized clinics affiliated to Jahrom University of Medical Sciences as patients’ companions and at least 6 months had passed from their last menstrual cycle. These individuals had no debilitating physical or mental disorders and expressed their menopausal experiences through semi-structured and unstructured interviews. In this study, the participants signed written consent form and were ascertained regarding anonymity and secrecy. They could also discontinue their cooperation with the researchers at any time.

### 2.1 Data Collection

Data were collected through semi-structured and sometimes unstructured interviews.

A pilot study was conducted to detect possible problems, and correct methodological errors (Polit, 2010). The pilot study was not used for this study.

In doing so, first general questions were asked and the interview was continued according to the participants’ responses. In this way, at first the participants were asked about their mentality regarding menopause. Then, more questions were asked about this period and their experiences of this evolutionary crisis and its related factors. It should be mentioned that the researcher controlled the accuracy of her interpretation of the participants’ responses using guide questions. The following questions were asked based on the participants’ experiences. The participants were also required to exemplify and provide reasons for their answers. The interviews lasted for 40-60 minutes.

### 2.2 Data Analysis

The data were analyzed through content analysis.

Content analysis is a specialized method for processing scientific data aims to provide cognition, new insight, picture of reality, guide for action, and interpretation of contextual information through a systematic process.

In doing so, the interview categorizations (Krippendorff, 2004; Babbie, 2010) were recorded, transcribed, encoded, and analyzed. Data analysis was performed simultaneously with data collection. The participants’ words and indicative codes (researcher’s interpretation) were used for primary encoding. Then, the next interviews were performed. The meaningful units were extracted from the participants’ words in form of primary or open codes. Afterwards, the codes were reviewed for several times and those which expressed a similar issue were classified in one category. This was continued and secondary encoding or data categorization was carried out. Then, the categories were compared and those which had similar features were combined. Finally, the themes were extracted.

### 2.3 Rigor

The validity was confirmed using participant and peer review. In order to assess the participants’ review, their words were returned and the codes and categories were given to 2 professors who were familiar with qualitative studies. For peer assessment also, the complete text, codes, and categories were presented to two faculty members and the opinions of two experts in qualitative research were asked.

It should be mentioned that a friendly atmosphere was created in the group and attempts were made to gain the participants’ consent for taking part in the group.

After investigating the opinions, two colleagues separately evaluated and matched the contents. Then, the categories and subcategories were identified. In order to enhance the reliability, the participants’ confirmation was also obtained.

## 3. Results

### 3.1 Qualitative Part

This study was conducted on 300 menopausal women who had referred to specialized clinics as patients’ companions. The frequency of psychosomatic disorders in these individuals is presented in Table 1. According to this table, the highest frequency of psychosomatic disorders was related to sexual disability 101 (33.7%), followed by hypertension 39 (13%) and constipation 30 (10%).

**Table 1:** Psychosomaticdisorders experienced by the study women (N=300)

Disorders	High	Average	Low	Asymptomatic
Headache	17 (5.6%)	46 (15.3%)	36 (12%)	99 (67%)
Muscle pain	3 (1%)	108 (36%)	36 (12%)	117 (39%)
Low appetite	20 (6.7%)	56 (18.7%)	63 (21%)	139 (46.3%)
Peptic ulcer	9 (3%)	8 (2.7%)	11 (3.7%)	272 (90.7%)
Chest pain	16 (5.3%)	33 (11%)	83 (27.7%)	169 (56.3%)
Constipation	30 (10%)	56 (18.7%)	19 (6.3%)	195 (65%)
Blood pressure	39 (13%)	58 (19.3%)	17 (5.7%)	114 (38%)
Rheumatism	1 (0.3%)	0	2 (0.6%)	297 (99.1%)
Eczema rash	8 (2.7%)	21 (7%)	78 (26%)	193 (64.3%)
Diarrhea	11 (3.7%)	26 (8.7%)	28 (9.3%)	235 (78.3%)
Hyperthyroidism	10 (3.4%)	1 (0.3%)	6 (2%)	283 (94.3%)
Shortness of breath	29 (9.7%)	39 (13%)	39 (13%)	193 (64.3%)
Irritable bowel	9 (3%)	6 (2%)	10 (3.3%)	275 (91.7%)
Sexual disability	101 (33.7%)	82 (27.3%)	24 (8%)	93 (31%)
Heart attack	10 (3.4%)	7 (2.4%)	17 (5.8%)	265 (88.4%)

The present study also utilized DSM IV criteria in order to determine hypochondria. The results revealed the rate of this disorder to be 2.9% among the study participants.

### 3.2 Qualitative Part

Out of 108 codes obtained in this study, 5 themes were identified which included change in emotion and mood (sensitivity, reduction of exhilaration, feeling of getting old, and tendency to be noticed), change in attitude toward fervency (reduction of capabilities, worrying about refractory diseases, reduction of physical strength, and negative attitude towards what others would say), change in self-concept (feeling of being different, change in beauty, and change in self-efficacy), and change in interpersonal relationships (getting distant from others, change in interaction with one’s husband).

**The first theme (change in emotion and mood)** involves impatience, reduction of exhilaration and vitality, sensitivity, and hiding one’s emotions. The participants believed that the change in their mood was quite obvious and prevented them from living their normal life. On the other hand, some others mentioned gentility and perfection as the main features of this period which every woman should be proud of. In this regard, one of the participants said: *“I expected less from life before. I didn’t take what others said serious. But it is not the case now. I immediately get upset. My children tell me that I am getting old and I am really sad. …”*. Another woman compared menopause with pre-menopause period and stated: *“Before menopause, I felt light when I was in my period. But it is not the case now. I feel heavy rather than comfortable and I can do nothing about it”*.

Change in emotions can affect these women’s behavior and performance and create changes in their relationships with their family members. For instance, one of the study participants mentioned: *“I feel weaker than before. I feel that I cannot do anything. …”*.

**The second theme was** **change in attitude and understanding**. This can present through change and reduction of capabilities, reduction of physical and mental power, and probability of the incidence of refractory diseases. Considering the reduction of mental strength, one of the study participants said: *“In the past, I knew everything and I quickly answered all the questions. But now, I feel slow. I am even slow in eating my food. I am impatient. I prefer to stay silent most of the time*”.

Furthermore, some women expressed their worry about suffering from refractory diseases and these negative mentalities obviously disturbed them.

Yet, some participants mentioned their binary feelings and attitudes towards menopause. In spite of counting positive features for menopause, they expressed their complaints and stated negative changes of this period. One woman said: *“We are villagers and do not know much. We just like to get rid of periods. Unlike those living in urban areas, we have not got facilities to care for ourselves. Tolerating periods was hard but now we are comfortable. …”*

Some study participants showed their worry about their health. They believed that menopause was the beginning of their physical and mental illness. One of the women mentioned: *“Menopause for me was accompanied by different disorders; hyperlipidemia, hot flashes. I am always receiving drugs. I just go to different doctors to see what the matter is with me”*.

Being worried about health, reduction of physical and mental strength, and change in attitude towards oneself and future are all associated with change in attitudes and understanding in this period.

Another theme of the study was **change in self-concept** which presents through regrets for youth, being different, change in appearance, and change in self-efficacy. One woman stated: *“I feel different from the past and this is hard. I feel aged. I have lost my vitality*”. Also, another participant said: *“Menopause is the beginning of getting old. But it is the will of God and we cannot stop it. I went to a doctor to stop it or experience lower complications, I even used herbal medication, but I could do nothing. I wish menopause had come to me later”*.

Another study theme was **change in interpersonal interactions** which was expressed through problems in relationships with others, getting distant from others, reduction of relationships, and change in relationships with one’s husband. Considering this issue, one of the study participants mentioned: *“I have lower capabilities. I rarely go out. I mostly like others to come and visit me”*.

In addition, some women stated that their husbands did not understand the process of menopause and change in the relationships between couples. For instance, one of the women said: *“Most often my husband does not understand my shortcoming and the fact that I am a menopausal woman. He does not understand what I mean when I say I am tired and it is time for me to rest*.….”

Negative thoughts resulting from couples’ cold relationships can sometimes be accompanied by stress. Besides, negative stress resulting from change in self-concept can increase the individuals’ depression, sadness, and disablement. In this regard, one of the study women said: *“I usually think now that I don’t have any desire, my husband may marry another woman. He is a man with strong desire but it’s not my fault. The thought that he is not interested in me anymore …”*.

Nonetheless, some women considered the reduction in sexual desire and relationship as the beginning of perfection. For instance, one participant stated: *“We are husband and wife and love each other but we have very few sexual relationships. We both feel old and our dignity should be maintained”*.

## 4. Discussion

The present study showed that, the rate of some psychosomatic disorders in menopause women was higher than others. Also hypochondrias reported 2.9% among the study women.

Women are considered as the basis of family health. In addition to the management of family members’ health, they play the main role in training and transferring healthy lifestyle to the next generation. Women in all age groups comprise a larger proportion of the population. However, their burden of disability is higher and they are encountered with particular problems resulting from their natural physiological status (Jones, 2003). In addition, they are faced with various problems, such as genitourinary, cardiovascular, neurological, and psycho-social problems, in this period (Speroff, 2005; Pesteei et al., 2008). These problems not only cause distress and disability for women, but they also impose a great pressure on the resources of health systems (Basu, 2004; Golyan Tehrani et al., 2002). This research confirmed our results in present study.

Other results in qualitative part showed that menopause women experience specific physical and mental changes which impose a great pressure on them.

Thus, being familiar with these changes and understanding their causes are highly necessary for any woman. This, in fact, helps women to enter menopause by sufficient knowledge and a positive attitude (Pesteei et al., 2008). Speroff (2005) believes that having knowledge about the physiological issues of menopause leads to development of a positive attitude toward this period. Although the present study women were not asked about the source of their information about menopause (whether they had asked someone or had just observed others’ complaints), studies by Sharon (2002) and Jamshidimanesh et al. (2009) showed that women had heard some undesirable signs from women around and subsequently had developed a negative attitude toward menopause. Negative attitudes of women in the present study were also in line with these two studies. On the other hand, a study conducted in the U.S. showed that most women felt comfortable with menopause and believed that they were a positive, experienced person (Avis & Mckinlay, 1991). Similarly, one study performed in India showed that these women considered menopause as a reward and a feeling of freedom (Punyahotra et al., 1997). Also, the study conducted by Donati et al. (2009) in Ecuador and the one performed by Leon et al. (2007) in Italy indicated that more than 90% of subjects considered menopause as a positive phenomenon and had a positive attitude toward this period. These results were confirmed in some parts of the present study.

According to Fishbein (1992) one of the problems that health workers face is the lack of balance between the women’s acceptance of menopause as a natural phenomenon and the healthcare which can prevent complications and chronic diseases in this period.

In the present study, 4 themes, including change in emotions and mood, change in attitude toward fervency, change in self-concept, and change in interpersonal relationships, were identified. In Hautman’s study (1996) conducted on 16 Filipino-American menopausal women, one main category (change in femininity) and 3 subcategories (living with physical changes, social changes, and change in relationships) were obtained. Moreover, George (2007) conducted a phenomenological research in order to investigate the experience of 15 American menopausal women. The main themes obtained in that study were expectations and realities, categorization of incidents, and a new life stage. The study results supported the complexity and uniqueness of menopause. Although menopause is common among women, each woman’s experience of menopause is as unique as her DNA. Jamshidimanesh (2009) also performed a study on 17 menopausal women and revealed two main themes of a physiological phenomenon and deprivation. In addition, the subcategories included waiting for experience, readiness for confrontation, and loss of femininity, youth, and a bright future. Although the participants of that study had accepted menopause as a natural phenomenon, they believed it caused the incidence of diseases, loss of youth, sadness, end of femininity, and end of hope for future. The themes of that study are similar to those of the current study which indicates negative attitudes of most menopausal women towards this period.

In another study, Walter (2000) assessed the experience of menopause in 21 women from the psychological point of view. According to results, women stated that they felt experienced; however, results showed the feeling of a lack of self-confidence and vulnerability to physical and emotional changes in these women, as well. They also believed that they had not been provided with sufficient information about this period. Although some women talked to their friends about menopause, most women were not willing to talk about this issue. This might be due to the negative view toward this natural phenomenon and considering this period as a sign for getting old, disability, and loss of strength and attractiveness. Our study also reported theses experiences.

Due to the fact that menopause has disturbed the feeling of being healthy; it causes great social and professional problems for women. Thus, it is necessary to perform medical interventions and make women familiar with this period in order to increase the time an individual can use one’s optimum physical, mental, and social activity and enhance their feeling of capability and satisfaction (Speroff, 2005; Kojori, 2009; Hellstrom et al., 2004; Quinn, 1991).

The first theme of the present study was ‘change in emotions and moods’. Depression is one of the most prevalent disorders among women. This indicates that most mental symptoms of this period are associated with psycho-social incidents, including change in relationship with the children, sexual status, and other life events (Speroff, 2005; Berek, 2007). These are also confirmed by the findings of the current study because the fourth theme, namely, ‘change in relationship with others’ and ‘change in interaction with one’s husband’, was accompanied by ‘change in moods’.

In an American study, Swan reported that the prevalence rate of ‘change in moods’ increased from 10% before menopause to 16.5% at the beginning of this period. Yet, some researchers believe these mood fluctuations to occur in response to hormonal changes near menopause (Speroff, 2005). According to the study by Bloch (2002), 35.3% of study subjects had mild depression. Moreover, the results of the study by Maziak et al. (2002) showed that depression was more prevalent among illiterate individuals. Besides, Hatice et al. (2008) reported that 29.3% of study participants were depressed and 65% suffered from sexual dysfunction.

Sexual activity is one of the main dimensions of quality of life which is affected by various factors. Sexual activity remains relatively constant in menopausal women and only half of these women continue their sexual activity (Speroff, 2005; Berek, 2007; Kojori, 2009). In general, sexual problems are common all through the reproductive ages; however, sexual dysfunction is quite more prevalent among menopausal women (Nappi & Lachowsky, 2009). The prevalence of sexual dysfunction in the U.S. has been reported to be 23-63% with the highest prevalence rate among menopausal women (Siarra et al., 2005; Murry, 2000). An Iranian study also indicated the prevalence rate of sexual dysfunction to be 72.4% among menopausal women (Beigi et al., 2008). On the other hand, the prevalence of this disorder was reported to be 20% in Bonnie’s (1999) study and 50% in Block’s (2002) study performed in the U.S.

According to the themes extracted in the present study, change in the women’s relationships with their husbands caused stress and eventually led to the development of a negative attitude toward menopause. Sexual dysfunction affects couples’ lives and influence marital relationships in particular (Castelo Branco, 2003). Considering the fact that many psychological and social problems in this period result from sexual disorders, specialists play a critical role in consultation and training in this regard (Beigi et al., 2008). Borrisova and Danacie (2003) conducted a study entitled “sexual desire and psychological changes in menopause” and stated that anxiety and depression had negative effects on sexual relationships. In the same line, Kingsberg (2002) showed that physical or mental problems and consequently reduction of desirable relationships with one’s sexual partner would lead to sexual disorders. Beutel (2002) also conducted a study on sexual desire and satisfaction with sexual relationships and stated that German old men believed appropriate relationship with spouse would increase sexual desire.

Another theme extracted from the current study was ‘change in attitude toward fervency’ which was expressed through reduction of capabilities, concern about refractory diseases, reduction of physical capability, and negative attitudes toward what others say. A review of literature shows that REM sleep is reduced in these ages. This implies that the proportion of the time one sleeps to the time one spends in bed decreases (Phillips & Ancoli, 2001). Insomnia affects the function of the nervous system and being awake for a long time is accompanied by progressive intellectual disorders (Hall, 2011). These might justify women’s experience of reduction of self-efficacy, mental and physical capabilities.

Based on the study by Fallahzadeh et al. (2011), memory disorder was among the 10 common complaints during menopause. Consistently, the findings of the study by Chim (2002) showed that 45.1% of study subjects suffered from memory loss and amnesia. Furthermore, being aware of the symptoms and complications of menopause can be effective in reducing the problems and increasing the quality of life in menopausal women. Rostami et al. (2003) and Moridi, Shohadaiee, and Abbassi (2005) indicated that health education changed women’s knowledge and attitude toward menopause and improved their quality of life. Women’s knowledge and belief in treatment methods led to reduction of menopause complications and development of a positive attitude toward oneself (Avis & Mckinlay, 1991).
